# High-resolution structures of AidH complexes provide insights into a novel catalytic mechanism for *N*-acyl homoserine lactonase

**DOI:** 10.1107/S0907444912042369

**Published:** 2012-12-20

**Authors:** Ang Gao, Gui-ying Mei, Shun Liu, Ping Wang, Qun Tang, Yan-ping Liu, Hui Wen, Xiao-min An, Li-qun Zhang, Xiao-xue Yan, Dong-cai Liang

**Affiliations:** a National Laboratory of Biomacromolecules, Institute of Biophysics, Chinese Academy of Sciences, Beijing 100101, People’s Republic of China; b Graduate University of Chinese Academy of Sciences, Beijing 100049, People’s Republic of China; c Department of Plant Pathology, China Agricultural University, Beijing 100193, People’s Republic of China

**Keywords:** quorum sensing, lactonases, α/β-hydrolases, acid–base catalysis, covalent catalysis

## Abstract

Many pathogenic bacteria that infect humans, animals and plants rely on a quorum-sensing (QS) system to produce virulence factors. *N*-Acyl homoserine lactones (AHLs) are the best-characterized cell–cell communication signals in QS. The concentration of AHL plays a key role in regulating the virulence-gene expression and essential biological functions of pathogenic bacteria. *N*-Acyl homoserine lactonases (AHL-lactonases) have important functions in decreasing pathogenicity by degrading AHLs. Here, structures of the AHL-lactonase from *Ochrobactrum* sp. (AidH) in complex with *N*-­hexanoyl homoserine lactone, *N*-hexanoyl homoserine and *N*-­butanoyl homoserine are reported. The high-resolution structures together with biochemical analyses reveal convincing details of AHL degradation. No metal ion is bound in the active site, which is different from other AHL-lactonases, which have a dual Lewis acid catalysis mechanism. AidH contains a substrate-binding tunnel between the core domain and the cap domain. The conformation of the tunnel entrance varies with the AHL acyl-chain length, which contributes to the binding promiscuity of AHL molecules in the active site. It also supports the biochemical result that AidH is a broad catalytic spectrum AHL-lactonase. Taken together, the present results reveal the catalytic mechanism of the metal-independent AHL-lactonase, which is a typical acid–base covalent catalysis.

## Abstract 2

Crystal structures of the AHL-lactonase AidH in complex with substrate and product are reported at high resolution and a catalytic mechanism is proposed for the metal-independent AHL-lactonase.

## 1. Introduction   

*N*-Acyl homoserine lactone (AHL) degrading enzymes degrade the AHL signal molecules of pathogens and break the quorum-sensing (QS) system to control pathogen infection. The QS system is a cell–cell communication mechanism in bacteria that is used to synthesize, secrete and detect small signal molecules in order to perceive the population density and regulate the expression of specific genes in response to a changing environment. More than 70 bacterial species are known to produce AHL-type signals. These signals are involved in the regulation of a range of important biological functions, including luminescence, antibiotic production, plasmid transfer, motility, virulence and biofilm formation (Dong & Zhang, 2005 ▶; Dong *et al.*, 2007 ▶). AHLs regulate virulence-gene expression in a range of plant and animal (including human) bacterial pathogens (Dong *et al.*, 2001 ▶), such as the plant pathogens *Agrobacterium tumefaciens* (Piper *et al.*, 1993 ▶) and *Erwinia carotovora* (Pirhonen *et al.*, 1993 ▶) and the animal pathogens *Pseudomonas aeruginosa* (Passador *et al.*, 1993 ▶) and *Burkholderia* (Ulrich, 2004 ▶). AHL-degrading enzymes have been utilized in the biocontrol of plant diseases because of their ability to degrade AHL signal molecules and to inhibit the virulence-gene expression of pathogens. For example, transgenic plants that express AHL-lactonase demonstrate an enhanced resistance to infection by *Pectobacterium carotovorum* subsp. *carotovorum* (Wang *et al.*, 2010 ▶).

To date, several AHL-degrading enzymes have been found in at least ten bacterial species. These enzymes can be divided into different types according to their catalytic sites. These enzymes include AHL-lactonase, which catalyzes AHL ring opening by hydrolyzing the ester bond of the lactone (Dong *et al.*, 2000 ▶), AHL-acylase, which hydrolyzes the amide linkage and separates the AHL into fatty acid and homoserine lactone (Huang *et al.*, 2003 ▶), AHL-oxidase, which catalyzes oxidation at the ω-1, ω-2 and ω-3 C atoms of the acyl chain of AHL (Chowdhary *et al.*, 2007 ▶), and so on. Among these AHL-degrading enzymes, AHL-lactonase is a hotspot in recent research. Phylogenetic analyses have shown that these prokaryotic AHL-lactonases can be grouped into AiiA-like and AttM-like clusters. Although the AHL-lactonases from the two clusters share less than 25% homology, both of the clusters contain a highly conserved motif H*X*H*X*DH that has proven to be essential for AHL-lactonase activity (Dong & Zhang, 2005 ▶; Wang *et al.*, 2010 ▶).

In 2005, the crystal structure of AHL-lactonase from *Bacillus thuringiensis* subsp. *kurstaki* (hereafter referred to as BTK-AiiA) was described at 1.6 and 2.0 Å resolution (Liu *et al.*, 2005 ▶; Kim *et al.*, 2005 ▶). In 2007, the structure of AHL-lactonase from *A. tumefaciens* (hereafter referred to as AiiB) was reported at 1.8 Å resolution (Liu *et al.*, 2007 ▶). Both BTK-AiiA and AiiB are members of the metallo-β-lactamase superfamily and their three-dimensional structures are very similar (Liu *et al.*, 2007 ▶). In both structures, two zinc ions are bound at the conserved H*X*H*X*DH motif and are located in a bridged dinuclear site (the active site), which plays a very crucial role in the catalytic reaction. The catalytic mechanism is considered to be dual Lewis acid catalysis (Liu *et al.*, 2005 ▶; Kim *et al.*, 2005 ▶).

In previous work (Mei *et al.*, 2010 ▶), we reported that AidH is a novel AHL-degrading enzyme from *Ochrobactrum*. High-performance liquid-chromatography (HPLC) and electrospray ionization mass-spectrometry (ESI-MS) analyses of the substrates and products indicated that AidH is an AHL-lactonase and catalyzes AHL ring opening by hydrolyzing lactones. The enzyme is a 251-residue protein and amino-acid sequence alignments reveal that it has only 8% identity to BTK-AiiA and 10% to AiiB (Supplementary Fig. S1^1^). Unlike most AHL-lactonases, AidH is proposed to be a member of the α/β-hydrolase fold family. In addition, it does not contain the conserved H*X*H*X*DH metal-binding motif. Moreover, Zn^2+^ is not essential for AidH activity. AidH is presumed to be a novel type of AHL-lactonase with a different catalytic mechanism to metal-dependent AHL-lactonases. However, the intriguing catalytic mechanism of these AHL-lactonases has not yet been elucidated. In this study, we determined the crystal structure of AidH at 1.29 Å resolution and the structures of AidH–C4-HS (*N*-­butanoyl homoserine), AidH_S102G_–C6-HSL (*N*-hexanoyl homoserine lactone) and AidH_E219G_–C6-HS (*N*-hexanoyl homoserine) complexes at resolutions of 1.09, 1.33 and 1.11 Å, respectively (Supplementary Fig. S2). Based on these structures, together with mutagenesis and biochemical data, we analyzed the active site of AidH and its binding to a substrate and to a product. We then proposed a catalytic mechanism for AHL-lactonase which differs from the previous model (Liu *et al.*, 2005 ▶; Kim *et al.*, 2005 ▶).

## 2. Materials and methods   

### 2.1. Protein expression and purification   

The *aidH* gene was cloned into the pET22b(+) expression vector with a C-terminal His tag. The vector was transformed into *Escherichia coli* BL21(DE3) cells. The cells were cultured in LB medium at 310 K until they reached log phase growth (OD_600_ = 0.6). AidH was induced by isopropyl β-d-1-thio­galactopyranoside at a final concentration of 0.8 m*M* at 301 K for 16 h according to the method described in previous work (Mei *et al.*, 2010 ▶). The cells were harvested, resuspended in lysis buffer (20 m*M* HEPES, 300 m*M* NaCl, 20 m*M* imidazole pH 7.0) and lysed by sonication. The lysate was clarified by centrifugation and purified using a nickel-affinity column. Further purification was performed *via* ion-exchange and size-exclusion chromatography on Resource Q and Superdex 200 (GE Healthcare) columns, respectively. The purified AidH was stored in 5 m*M* Tris–HCl, 300 m*M* NaCl pH 8.0 at a concentration of 30 mg ml^−1^ for crystallization.

### 2.2. Site-directed mutagenesis   

Site-directed mutagenesis was performed using a QuikChange site-directed mutagenesis kit (Stratagene). Plasmid pET22b(+)-*aidH* was used as the template. All mutations were confirmed by sequencing. The expression and purification of the mutant variants were performed in the same way as for wild-type AidH.

### 2.3. Crystallization and data collection   

Crystals of AidH were grown at 293 K using the hanging-drop vapour-diffusion method. Drops consisted of 2 µl each of the protein and reservoir solutions. The reservoir solution for the crystallization of wild-type AidH consisted of 25% PEG 8000, 0.2 *M* ammonium acetate, 0.01 *M* magnesium acetate, 0.05 *M* sodium cacodylate pH 6.5. The reservoir solution for AidH_S102G_ consisted of 30% PEG 8000, 0.2 *M* lithium acetate, 0.1 *M* sodium acetate pH 5.6. The reservoir solution for AidH_E219G_ consisted of 35% PEG 8000, 0.2 *M* lithium acetate, 0.1 *M* sodium acetate pH 6.3. Pt derivatives were obtained by soaking the native crystals in reservoir solution supplemented with 10 m*M* K_2_PtCl_4_ for 12 h. Crystals of AidH complexed with AHL (or *N*-acyl homoserine; AHS) were obtained by soaking. The crystals were soaked in reservoir solution containing an additional 100 m*M* AHL for between 10 s and 1 h. Native and derivative crystals were soaked in 15% glycerol for 2 min and were flash-cooled in liquid nitrogen before data collection. Diffraction data sets for wild-type AidH and the Pt derivative were collected on beamline BL-17A (Photon Factory, KEK, Japan) and in the IBP core facility centre (Institute of Biophysics, Chinese Academy of Sciences, People’s Republic of China), respectively. Data sets for free-form AidH_S102G_ and AidH_E219G_ were collected on beamline BL44XU at SPring-8, Japan. Data sets for all of the complexes were collected on beamline BL-17U at SSRF, People’s Republic of China. All data sets were integrated and scaled with *HKL*-2000 (Otwinowski & Minor, 1997 ▶). A statistical analysis of the data collection is summarized in Table 1 ▶.

### 2.4. Structure determination and refinement   

The program *AutoSol* in the *PHENIX* software package (Adams *et al.*, 2010 ▶) was used to calculate the preliminary phase angles of wild-type AidH by single-wavelength anomalous diffraction (SAD). Using the initial phase from *PHENIX*, an automatic model was built with *ARP*/*wARP* (Perrakis *et al.*, 1999 ▶) at 1.29 Å resolution. Model refinement was performed in *REFMAC*5 (Murshudov *et al.*, 2011 ▶) and *PHENIX*. The program *Coot* (Emsley & Cowtan, 2004 ▶) was used for inspection and manual improvement of the model. The final model included 550 amino-acid residues and 929 water molecules. Within the resolution range 20–1.29 Å, the native structure was refined to a final *R*_work_ of 12.28% and *R*_free_ of 15.70%. Acceptable stereochemistry was confirmed using a Ramachandran plot calculated by *PROCHECK* (Laskowski *et al.*, 1993 ▶). The structures of the AidH_S102G_–C6-HSL complex, the AidH–C4-HS complex, the AidH_E219G_–C6-HS complex, AidH_S102G_ and AidH_E219G_ were determined by molecular replacement employing wild-type AidH as the model and were refined as described above for wild-type AidH. The statistics of the refinement and the stereochemistry of the final models are summarized in Table 1 ▶. The coordinates and structure factors were deposited in the Protein Data Bank under accession codes 4g5x, 4g9e, 4g8d, 4g8b, 4g9g and 4g8c, respectively.

### 2.5. HPLC/ESI-MS analysis   

To determine how AidH inactivates AHL signals, we analyzed the reaction substrate and product by HPLC and ESI-MS following the procedure described in previous work (Mei *et al.*, 2010 ▶). The experiment was performed using a single-quadrupole LC/MS system (Agilent 6110). We mixed wild-type AidH (at a final concentration of 100 µg ml^−1^) with AHL (at a final concentration of 1 m*M*) in reaction buffer (50 m*M* Tris–HCl, 400 m*M* NaCl pH 7.0). After incubation at 310 K for 30 min, the mixture was evaporated on a rotary evaporator. For HPLC analysis, the sample was redissolved in 0.1 ml methanol and analyzed using a Symmetry C18 reverse-phase column (4.6 × 150 mm; Agilent TC-18). For AHLs with different hydrophobicities, we optimized the components and the flow rate of the mobile phase for the different types of AHLs.

### 2.6. Enzyme assay   

The enzyme assay was performed as described previously (Mei *et al.*, 2010 ▶). The protein was mixed with the substrate in reaction buffer (50 m*M* Tris–HCl, 400 m*M* NaCl pH 7.0) and incubated at 310 K for 15 min. Subsequently, the sample was evaporated by vacuum evaporation and redissolved in methanol. The redissolved sample (1 µl) was then cultured with 0.3 ml of the AHL biosensor *A. tumefaciens* NTL4 (pZLR4) at 301 K for 3 h. The β-galactosidase expressed by the biosensor cells was determined using the method of Miller (1972 ▶). The residual AHLs were also detected using agar plates. ABM agar (0.6%) containing 5 ml exponentially grown *A. tumefaciens* NTL4 (pZLR4) and X-Gal (40 µg ml^−1^) was poured into the plates and the AHL samples were applied onto the plates. The plates were incubated overnight at 301 K. The AHL activities were determined by the appearance of blue spots on the plates.

## 3. Results   

### 3.1. Overall structure of AidH   

**Figure 1:**
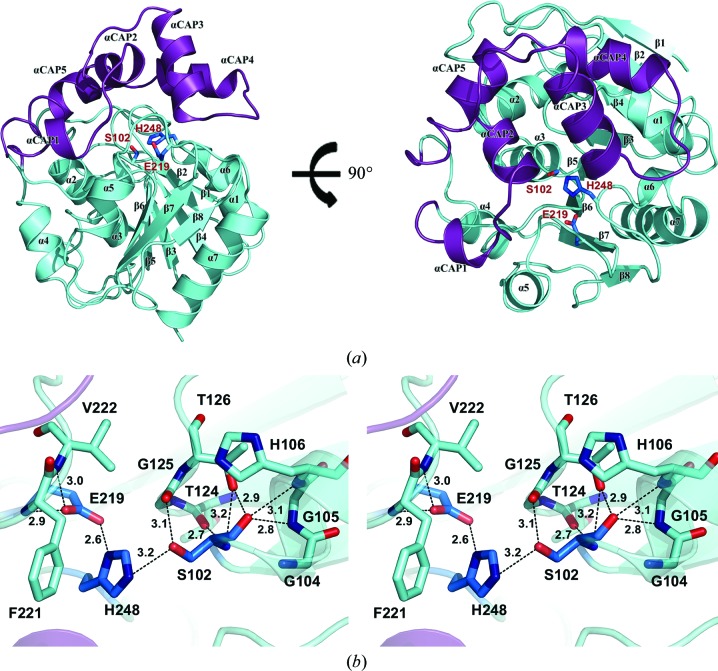
Overall structure and active site of AidH. (*a*) Top and side views of the AidH monomer. Cartoon representation of AidH with the α/β-hydrolase fold core domain and the cap domain shown in aquamarine and violet/purple, respectively. Secondary-structure elements and catalytic triad residues are labelled in black and red, respectively. (*b*) Wall-eyed stereo presentation of the active site of AidH. The active site of AidH is the conserved serine protease-type catalytic triad: Ser102, His248 and Glu219. C atoms of the catalytic triad residues and other residues are shown in marine and pale cyan, respectively. O atoms and N atoms are shown in red and deep blue, respectively. Hydrogen bonds are depicted as black short-dashed lines. Interatomic distances are shown in Å.

The crystal structure of AidH was determined at 1.29 Å resolution using single-wavelength anomalous dispersion (SAD) phasing. The statistics of data collection and refinement are presented in Table 1 ▶. The crystal has the symmetry of space group *P*2_1_ and contains two molecules per asymmetric unit. The surface area between the two molecules in the same asymmetric unit is 959 Å^2^, which is 9% of the total surface area of a molecule (10 636 Å^2^). Size-exclusion chromatography and analytical ultracentrifugation data for AidH indicate that it is monomeric in solution. Furthermore, in the structure of the complex of AidH and its substrate C6-HSL, each AidH molecule binds one substrate molecule in the active site. Therefore, the functional unit of the enzyme is a monomer and the dimeric organization on crystallization is likely to be an artifact of the crystal packing. Based on a topological comparison (Bond, 2003 ▶; Supplementary Fig. S3) and a *DALI* search (Holm & Sander, 1993 ▶), AidH is most similar in three-dimensional structure to a bromoperoxidase from *Bacillus anthracis* (PDB entry 3fob; Center for Structural Genomics of Infectious Diseases, unpublished work) and an esterase from *Pseudomonas putida* (PDB entry 1zoi; Elmi *et al.*, 2005 ▶), with *Z*-scores of 28.1 and 27.7, respectively, indicating that AidH is a member of the α/β-hydrolase family like 3fob and 1zoi. The overall structure of AidH has a two-domain architecture consisting of a typical α/β-hydrolase fold core domain capped by a cap domain (Fig. 1 ▶ *a*). The α/β-hydrolase fold core domain consists of an eight-stranded mostly parallel β-sheet (only the second β-strand, β2, is antiparallel to the rest) surrounded by seven α-­helices. The β-strands display a left-handed twist, with the first and last strands being oriented approximately perpendicular to each other. The seven α-helices are divided into two groups. α1, α6 and α7 are located on one side of the β-­sheet, whereas α2, α3, α4 and α5 are on the other side of the β-sheet. The cap domain composed of five α-helices is inserted between strands β6 and α4 of the core domain. The α/β-hydrolase fold domain and the cap domain are linked by loop 125–133 and loop 190–199.

In AidH, the classic catalytic triad Ser102/His248/Glu219 (Fig. 1 ▶ *b*) of the α/β-hydrolase fold superfamily is located at the surface between the core and cap domains. The whole active site is covered by the cap domain. The catalytic nucleophile residue Ser102 protrudes from the nucleophile elbow, which is a tight turn between β5 and α3. The conserved motif G*X*S/D/C*X*G in α/β-hydrolases allows efficient presentation of serine at a suitable position for attack on the substrate. Glu219 is located in the loop that follows β7 and is proposed to be the acidic member of the catalytic triad. The carboxyl O atom O^∊1^ of Glu219 forms a short and very strong low-barrier hydrogen bond (LBHB; Holmquist, 2000 ▶) to the N^δ1^ atom of His248. This interaction will stabilize the positive charge on His248 and hence increase the reactivity of the nucleophile Ser102. His248 is located in the loop between β8 and the following helix α6. The N^∊2^ atom is hydrogen-bonded to the hydroxyl O atom of Ser102, whereas the N^δ1^ atom forms a hydrogen bond to Glu219.

Accurate electron-density maps indicate the absence of metal ions or ligands in the active site, in contrast to BTK-AiiA (PDB entry 2a7m; Liu *et al.*, 2005 ▶) and AiiB (PDB entry 2r2d; Liu *et al.*, 2007 ▶). Using the *DALI* server (Holm & Sander, 1993 ▶), we compared the AidH structure with the structures of BTK-AiiA and AiiB. The results indicate significant structural differences between AidH and the other two enzymes, with *Z*-scores of 0.3 and 0.5.

### 3.2. Substrate binding   

**Figure 2:**
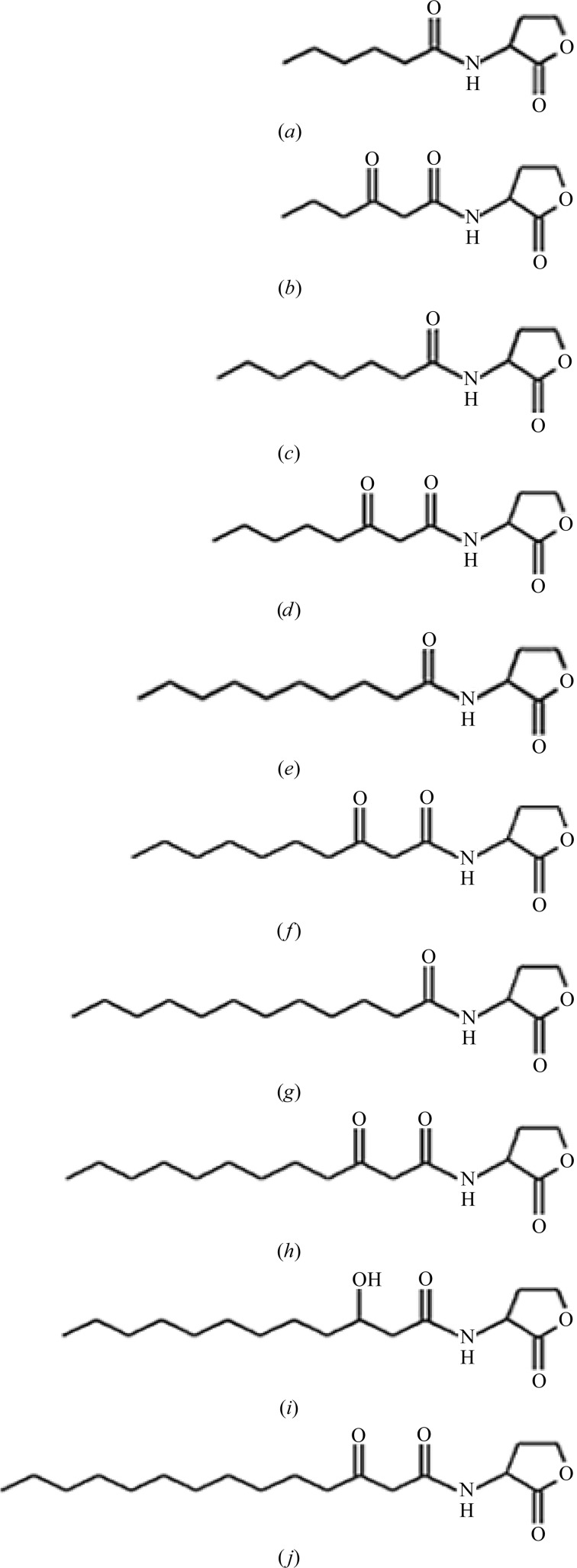
Structures of the substrates in Table 2 ▶.

**Figure 3:**
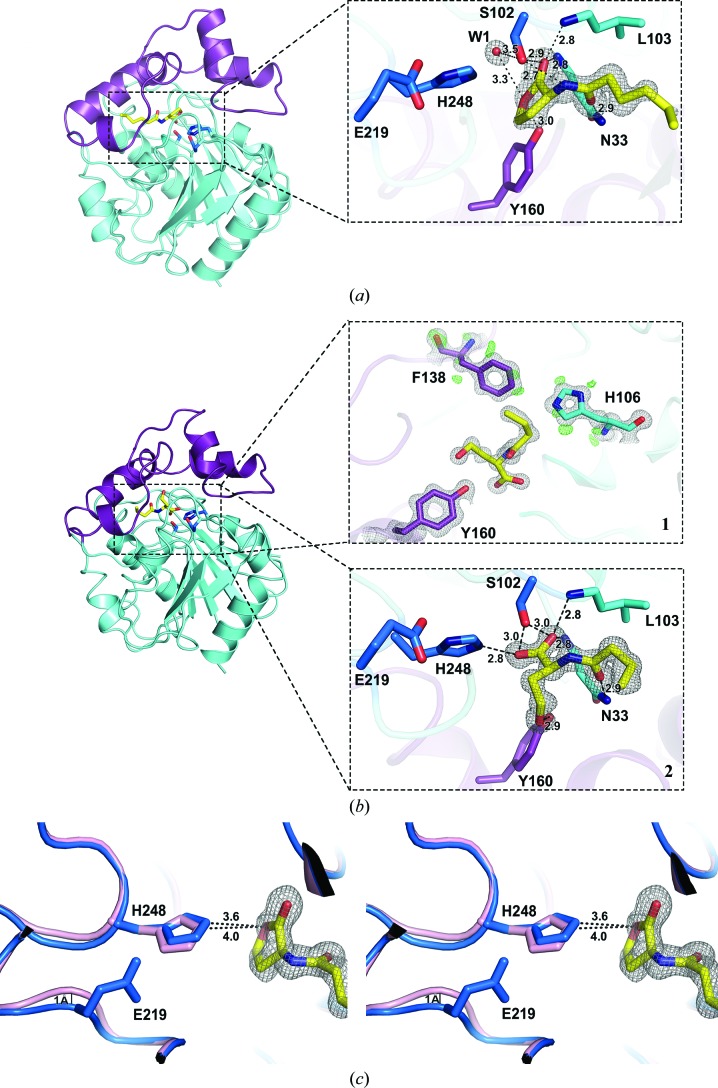
Substrate and product binding to AidH. (*a*) Active site of the AidH_S102G_–C6-HSL (enzyme–substrate) complex. The substrate C6-HSL binds to AidH_S102G_ by hydrogen-bonding interactions with the residues in the active site. The water molecule W1 close to the substrate is indicated as a red sphere. (*b*) Active site of the wild-type AidH–C4-HS (enzyme–product) complex. The electron density of some H atoms (shown as a green mesh) can be observed in panel 1. In panel 2, the product C4-HS binds in the active site of AidH. In (*a*) and (*b*), the 2*F*_o_ − *F*_c_ electron density for the bound C6-HSL and C4-HS contoured at the 1.5σ level is shown as a grey mesh. C6-HSL and C4-HS are indicated in yellow stick representation. (*c*) Wall-eyed stereo presentation of the superimposed active sites of the AidH_S102G_–C6-HSL complex and the AidH_E219G_–C6-HS complex, revealing the positional divergence in His248 and the loop where Glu219 is located. Residues of the AidH_S102G_–C6-HSL complex and AidH_E219G_–C6-HS complex are shown in marine and pink, respectively.

The ability of AidH to degrade a range of AHLs with different acyl-chain lengths and substituents at the C3 position was determined. No obvious difference in degrading activity was found towards these varied substrates (Table 2 ▶ *a*; Fig. 2 ▶). Furthermore, we digested AHLs with the enzyme and analyzed the reaction products by HPLC and ESI-MS. The enzymatic action on AHLs leads to a mass increase of 18 in the product, corresponding to a water molecule (Supplementary Fig. S4). This finding is in agreement with the chemical composition of lactone-opened AHLs. These results demonstrate that AidH is an AHL-lactonase that hydrolyzes the ester bond of the homoserine lactone ring of AHLs (the scheme of the reaction catalyzed by AHL-lactonase is shown in Supplementary Fig. S5). In addition, it has no acyl-chain length or C3 substituent preference and exhibits a broad catalytic spectrum. To assess the roles of the selected residues in catalysis, we performed a mutational analysis. According to the three-dimensional structure of AidH, the catalytic triad and the residues surrounding the active site (Asn33, Met77, Ser102, Leu103, Phe138, Met144 ,Tyr160, Met188, Phe189, Phe192, Glu219, Phe221 and His248) were mutated to glycine by site-directed mutagenesis and the activities of these protein variants towards 3-oxo-C8-HSL and C12-HSL were measured (Table 2 ▶ *b* and Supplementary Fig. S6). Conforming to our predictions, the catalytic triad mutations S102G and H248G disabled AidH from degrading AHLs. Surprisingly, the mutant AidH_E219G_ is not completely inactive and the activity of AidH_Y160G_ is significantly decreased. We further determined the crystal structure of the inactive mutant AidH_S102G_ in complex with C6-HSL (AidH_S102G_–C6-HSL). The crystal structure at high resolution (1.33 Å) revealed the first step of the catalytic reaction, *i.e.* substrate binding to the enzyme at the active site (Fig. 3 ▶ *a* and Supplementary Figs. S9*a* and S10*a*). C6-HSL is fastened in the active site of AidH by a combination of hydrophobic interactions and hydrogen bonds (Fig. 3 ▶ *a* and Supplementary Fig. S7). The carbonyl O atom in the C6-HSL lactone ring forms hydrogen bonds to the backbones of Asn33 and Leu103, and the O^δ1^ atom interacts with the hydroxyl O atom of Tyr160 *via* a hydrogen bond. To evaluate the role of the nucleophile Ser102 of the catalytic triad in substrate binding, we modelled a hydroxyl group on the side chain of the mutated Gly102 according to the conformation of Ser102 in wild-type AidH. The hydroxyl O atom of Ser102 forms hydrogen bonds to the carbonyl O atom of the lactone ring of the substrate and the N atom of the acyl chain of the substrate with lengths of 2.89 and 2.67 Å, respectively. The distance between the hydroxyl O atom of Ser102 and the carbonyl C atom of the C6-HSL lactone ring (2.52 Å) suggests that this C atom is likely to be the target when the nucleophile Ser102 attacks the substrate. In addition, one water molecule, W1, is close to the lactone ring of the substrate and Ser102 (Fig. 3 ▶ *a*), suggesting that this water molecule could act as the nucleophile in the hydrolysis of the substrate.

### 3.3. Product binding   

The crystal structure of wild-type AidH complexed with the product C4-HS (AidH–C4-HS) was determined at 1.09 Å resolution. Electron densities of some H atoms could be observed in this high-precision structure (panel 1 of Fig. 3 ▶ *b*). The accurate electron-density maps allowed the unambiguous modelling of all atoms involved in the product–enzyme complex (panel 2 of Fig. 3 ▶ *b*; Supplementary Figs. S9*b* and S10*b*). Following the lactone-bond breakage of *N*-butanoyl homoserine lactone (C4-HSL), the reaction product C4-HS features newly formed hydroxyl and carboxyl groups. The C4-­HS contains the O atom derived from the water molecule. The new carboxyl group forms hydrogen bonds to Ser102 and His248. In addition, the new hydroxyl group shows a rotation of approximately 98° around the axis of the C—C bond, resulting in a stretched conformation, and perfectly stabilizes this conformation by forming a hydrogen bond to Tyr160, which shifts by 1.3 Å compared with the substrate–enzyme complex. Tyr160 clutches the substrate and the product at the active site and positions them in the correct conformation *via* hydrogen bonds. Moreover, biochemical analysis indicates that replacement of Tyr160 has a strong impact on its catalytic efficiency. In addition to the catalytic triad residues, Tyr160 is suggested to play a key role in catalysis. We also solved the crystal structure of AidH_E219G_–C6-HS at 1.11 Å resolution. When AidH_E219G_ crystals are soaked with C6-HSL, C6-HSL is degraded to C6-HS, indicating that the AidH_E219G_ mutant does not lose its hydrolytic activity completely. The absence of the Glu219 side chain causes the backbone of the loop between β7 and α5 to shift by 1 Å. Furthermore, the loss of the carboxyl group of Glu219 breaks the hydrogen bond between Glu219 and the N^δ1^ atom of His248 and then causes a conformational change of His248 that distances it from the substrate (Fig. 3 ▶ *c*). Although the conformational change of His248 reduces the hydrolytic efficiency, AidH_E219G_ is not completely inactive. This result is consistent with the results of the activity assay (Table 2 ▶ *b* and Supplementary Fig. S6). Moreover, the hydroxyl group in the structure of AidH_E219G_–C6-HS flips to the opposite side compared with that in AidH–C4-­HS and forms a hydrogen bond to Met144 instead of Tyr160 (Supplementary Fig. S8). Two possible conformations of the newly formed hydroxyl group are found. The two or three terminal C atoms of the acyl chain in the AidH_E219G_–C6-HS structure shift by approximately 3 Å compared with AidH_S102G_–C6-HSL. The low-quality electron density and higher *B* factor (31.5 Å^2^) in this region indicate that the AHL tail is relatively flexible.

### 3.4. Substrate-binding tunnel   

**Figure 4:**
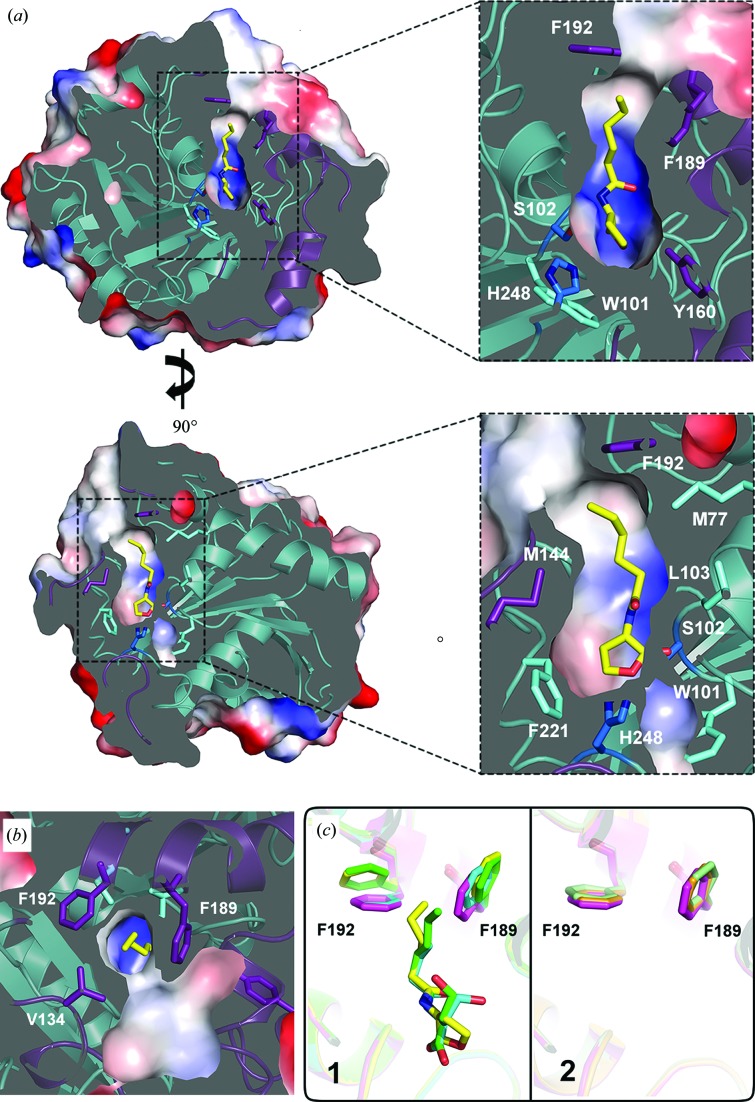
Substrate-binding tunnel of AidH and the conformational change of the tunnel entrance. (*a*) Sliced-surface view of the substrate-binding tunnel. The tunnel lying between the core and cap domains is lined by hydrophobic residues and has an overall positive charge. (*b*) The entrance of the tunnel is located on the cap domain and surrounded by hydrophobic residues. In (*a*) and (*b*), the molecular surface is coloured according to the electrostatic potential. Positive and negative potentials are shown in blue and red, respectively. (*c*) The distance between Phe189 and Phe192 changes upon substrate/product binding. In panel 1, free AidH, C4-bonded AidH and C6-bonded AidH_S102G_ (and AidH_E219G_) are shown in purple, cyan and yellow (and green), respectively. Panel 2 shows that there is no distance change in free-form AidH_S102G_ (orange) and AidH_E219G_ (grey).

Based on the high-precision complex structures, we found an approximately 14 Å long narrow tunnel that connects the bulk solvent and the active site through which the substrates access and bind to the active site (Fig. 4 ▶ *a*). The tunnel lies between the core and cap domains and is formed by the juxtaposition of these two domains. The entrance to the tunnel is located on the cap domain and is surrounded by the hydrophobic residues Val134, Met144, Phe189 and Phe192 (Figs. 4 ▶ *a* and 4 ▶ *b*). The tunnel wall is lined by the cap-domain residues Phe138, Met144 and Met188 as well as the core-domain residues Met77, Leu103 and His106. The end of the tunnel is a cavity with a diameter of 6 Å in which catalysis occurs. In addition to the catalytic triad residues, several aromatic residues (Trp101, Tyr160 and Phe221) are also found in this active site. The tunnel has an overall positive charge, so that the negatively charged substrate will be drawn toward the catalytic triad. Based on the crystal structures, we mutated the residues that constitute the substrate-binding tunnel to glycine. Mutation of the hydrophobic residues M188G, F189G, F192G and F221G decreases the activity to a certain degree (Table 2 ▶ *b* and Supplementary Fig. S6). We believe that the mutation of these hydrophobic residues, which decreases the hydrophobicity of the substrate-binding tunnel, will affect the binding affinity of the substrate. The point mutation of Tyr160 in the cap domain significantly decreases the activity, suggesting that this residue plays an important role in substrate binding and catalysis. It is noteworthy that the distance between Phe189 and Phe192, which surround the entrance of the substrate-binding tunnel, increases by approximately 2 Å upon substrate (or product) binding compared with the free wild-type AidH structure (panel 1 of Fig. 4 ▶ *c*). However, in the structures of free-form AidH_S102G_ and AidH_E219G_ the distance does not change (panel 2 of Fig. 4 ▶ *c*). This indicates that the conformational change in the tunnel entrance is the result of substrate or product binding. The entrance to the substrate-binding tunnel is in the open state upon substrate or product binding. Moreover, the size of the entrance varies with different substrate acyl-chain lengths. Compared with C4-HSL, the entrance expands more when C6-HSL (or C6-HS) binds to AidH (panel 1 of Fig. 4 ▶ *c*). Therefore, the entrance of the tunnel is proposed to undergo a closed–open–closed structural rearrangement during substrate binding and to accommodate the degree of closure to different substrates. The tunnel participates in all aspects of substrate selection, substrate binding, catalysis and product release, and plays an important role in the enzymatic reaction.

## 4. Discussion   

**Figure 5:**
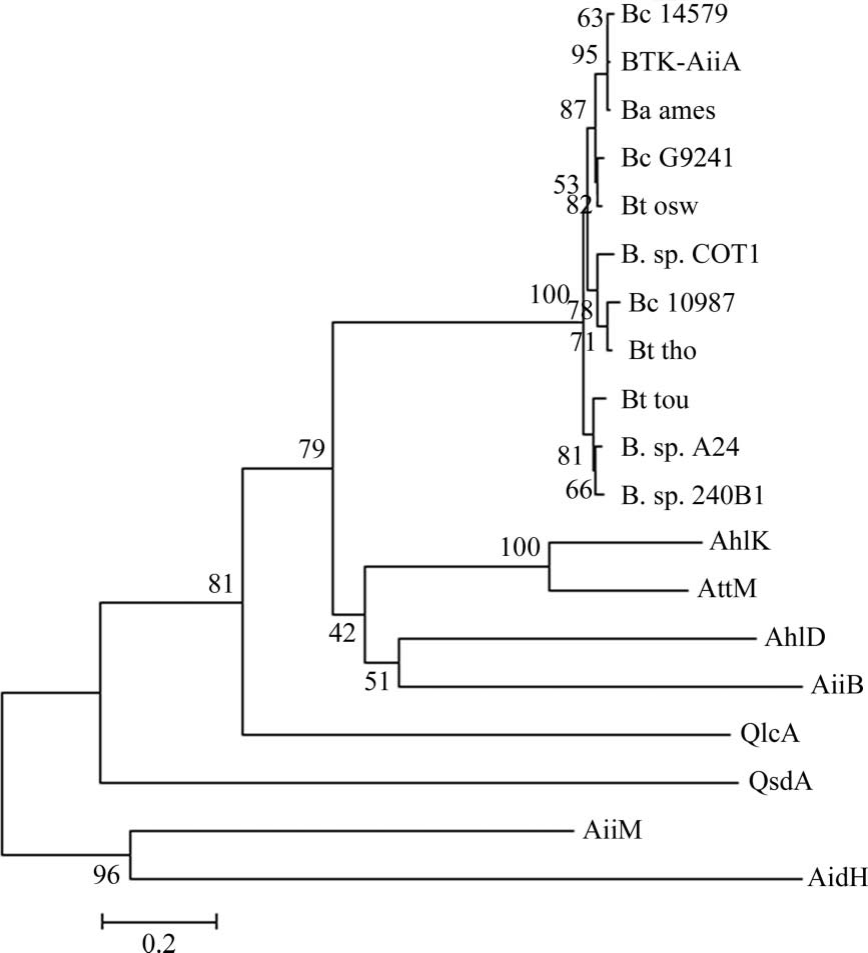
Phylogenetic analysis of AHL-lactonases. The lactonases (from top to bottom) are from *B. cereus* 14579 (Bc 14579; gi:30021556), *B. thuringiensis* subsp. *kurstaki* HD263 (BTK-AiiA; gi:22095303), *B. anthracis* Ames strain (Ba ames; gi:30263417), *B. cereus* G9241 (Bc G9241; gi:47564581), *B. thuringiensis* serovar oswaldocruzi (Bt osw; gi:28413776), *Bacillus* sp. COT1 (B. sp. COT1; gi:19773593), *B. cereus* ATCC10987 (Bc 10987; gi:42738443), *B. thuringiensis* serovar thompsoni (Bt tho; gi:22095299), *B. thuringiensis* serovar toumanoffi (Bt tou; gi:22095301), *Bacillus* sp. A24 (B. sp. A24; gi:21541343), *Bacillus sp*. 240B1 (B. sp. 240B1; gi:7416989), *Klebsiella pneumoniae* (AhlK; gi:31540969), *A. tumefaciens* (AttM; gi:17223785), *Arthrobacter* sp. IBN110 (AhlD; gi3:1580543), *A. tume­faciens* (AiiB; gi:16119885), uncultured Acidobacteria bacterium cosmid p2H8 (QlcA; gi:157644500), *Rhodococcus* sp. MP50 (QsdA; gi:146742384), *M. testaceum* StLB037 (AiiM; gi:334302761) and *Ochrobactrum* sp. strain T63 (AidH; gi:270313530).

In 2010, we reported that AidH is a novel AHL-lactonase (Mei *et al.*, 2010 ▶). AidH is a member of the α/β-hydrolase superfamily and has less than 21% identity to the other known AHL-lactonases, which are BTK-AiiA from *B. thuringiensis* subsp. *kurstaki* HD263 (8% identity; Liu *et al.*, 2005 ▶), AiiB from *A. tumefaciens* (10% identity; Liu *et al.*, 2007 ▶), AttM from *A. tumefaciens* C58 (7% identity; Zhang *et al.*, 2002 ▶), AiiM from *Microbacterium testaceum* (12% identity; Wang *et al.*, 2010 ▶), AhlD from *Arthrobacter* sp. IBN110 (11% identity; Park *et al.*, 2003 ▶), QsdA from *Rhodococcus erythropolis* W2 (21% identity; Uroz *et al.*, 2008 ▶) and QlcA from the soil metagenome (14.9% identity; Riaz *et al.*, 2008 ▶). A phylo­genetic tree of the representative AHL-lactonase was constructed (Fig. 5 ▶). Phylogenetic analysis shows that AHL-lactonases can be divided into group A and group B. Members of group A include the AiiA cluster, the AttM cluster and QsdA, which are all metal-dependent proteins. The AiiA and AttM clusters are members of the metallo-β-lactamase superfamily and contain a highly conserved H*X*H*X*DH metal-binding motif. The QsdA protein belongs to the phosphotriesterase family, another zinc-dependent metalloprotein family, and contains two conserved zinc-binding domains. The members of the AiiA cluster share more than 90% peptide sequence identity and less than 25% homology with AttM-cluster members (Dong & Zhang, 2005 ▶). Group B contains two members: AidH from *Ochrobactrum* sp. strain T63 and AiiM from *M. testaceum* StLB037 (Wang *et al.*, 2010 ▶). They are members of the α/β-hydrolase superfamily and do not contain a metal-binding motif. Moreover, metal ions are not required for their AHL-degrading activity. The three-dimensional structures of AHL-lactonase reported to date are those of BTK-AiiA and AiiB. Both of them are members of the metallo-β-lactamase superfamily and belong to group A, whereas AidH is a member of the α/β-hydrolase superfamily and belongs to group B. The three-dimensional structures of AidH and the other two proteins are totally different (*Z*-scores of 0.3 and 0.5). The results of both sequence alignment and structure comparison demonstrate that AidH is a novel AHL-lactonase. AidH must have a catalytic mechanism for AHL degradation that differs from that of BTK-AiiA and AiiB.

**Figure 6:**
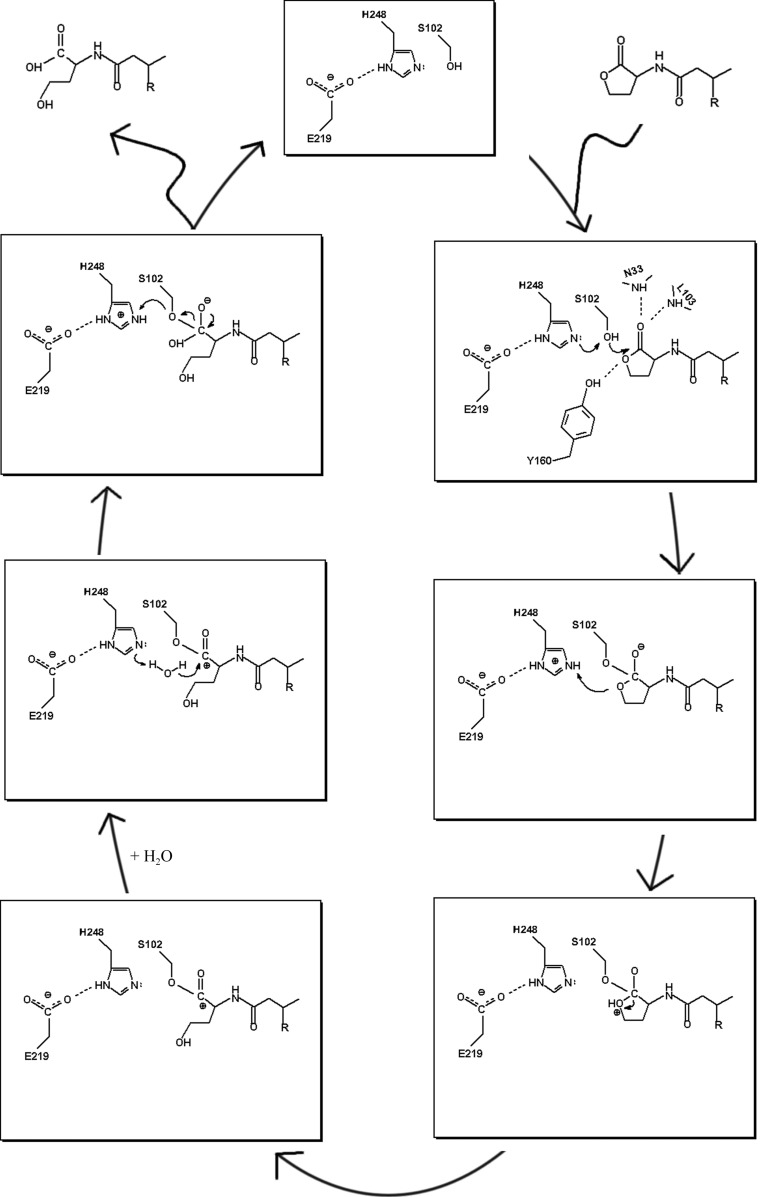
Proposed reaction mechanism of the degradation of AHLs catalyzed by AidH.

Based on three-dimensional structures and biochemical data, we propose a catalytic mechanism for the AHL degradation of the metal-independent AHL-lactonase (Fig. 6 ▶). (i) The structure of AidH shows that it possesses a conserved catalytic triad (Ser102, His248 and Glu219) and a recognizable substrate-binding tunnel. When the residues Phe189 and Phe192 surrounding the entrance of the tunnel open, the substrate reaches the triad *via* the narrow passage and binds in the active site by a combination of hydrophobic interactions and hydrogen bonds. The carbonyl O atom of the lactone ring of the substrate is hydrogen bonded to the backbone of Asn33 and Leu103, and the O^δ1^ atom of the lactone ring of the substrate interacts with the hydroxyl O atom of Tyr160 *via* a hydrogen bond. As a result, the lactone ring of the substrate is protonated. A hydrogen-bond network connects Ser102 to Glu219 *via* His248. His248 acts as a powerful general base and abstracts a proton from Ser102 to activate it as a nucleophile. In this process, the His-Asp charge-relay dyad mediates proton abstraction and makes the N atom of His248 much more electronegative. The biochemical data demonstrate that the Glu219→Gly mutation weakens the His-dependent solvent-assisted deprotonation and thus decreases the activity of AidH. (ii) The hydroxyl O atom of Ser102 is close to the carbonyl C atom of the lactone ring of the substrate (2.52 Å), so that the deprotonated Ser102 acting as a nucleophile conveniently attacks the carbonyl C atom of the substrate and forces His248 to accept a hydrogen and become protonated. As a result, an enzyme–substrate tetrahedral intermediate is generated. The tetrahedral intermediate is negatively charged and this negative charge is stabilized by the oxyanion hole. (iii) As a proton donor, His248 donates a proton to the O^δ1^ atom of the lactone ring and an unstable transition form is generated. The protonation of O^δ1^ considerably weakens the bond joining the C atom and the O atom in the ring. (iv) The C—O bond is subsequently broken. (v) The deprotonated substrate represents the pressing need to bring a water molecule into the reaction. One water molecule, W1, close to the lactone ring of the substrate is present in the structure of the AidH–substrate complex. As a general base, His248 deprotonates the water molecule. The remaining OH^−^ of the water attacks the carbonyl C atom as a nucleophile and another unstable intermediate is formed. (vi) In the final step of the reaction, the intermediate collapses and Ser102 abstracts a proton from the protonated His248. As a result, the product is ejected and the enzyme is regenerated for a new catalytic cycle. Throughout the reaction, the carboxyl group of Glu219 forms a hydrogen bond to His248, giving it the correct conformation and the proper tautomeric equilibrium after it accepts the proton from Ser102. The catalytic mechanism of AidH is classified as a typical acid–base catalysis and covalent catalysis.

Based on the structures of the AHL-lactonase complexes and the results of the biochemical assay, we propose that the catalytic mechanism of AHL-lactonase AidH is metal-independent. AidH can degrade a range of AHLs and has a broad catalytic spectrum. This study will help us to thoroughly understand the novel catalytic mechanism of AHL degradation and should be important in developing therapeutic strategies for the control and prevention of infectious bacterial diseases.

## Supplementary Material

PDB reference: AidH, 4g5x

PDB reference: complex with C4-HS, 4g9e

PDB reference: S102G mutant, 4g8d

PDB reference: S102G mutant, complex with C6-HSL, 4g8b

PDB reference: E219G mutant, 4g9g

PDB reference: E219G mutant, complex with C6-HS, 4g8c

Supporting information file. DOI: 10.1107/S0907444912042369/dw5029sup1.pdf

## Figures and Tables

**Table 1:** Data-collection and refinement statistics Values in parentheses are for the highest resolution shell.

	AidH	AidH (SAD)	AidHC4-HS	AidH_S102G_C6-HSL	AidH_E219G_C6-HS	AidH_S102G_	AidH_E219G_
Data collection							
Space group	*P*2_1_	*P*2_1_	*P*2_1_	*P*2_1_	*P*2_1_	*P*2_1_	*P*2_1_
Unit-cell parameters							
*a* ()	42.4	42.4	42.2	42.3	42.4	42.4	42.4
*b* ()	129.5	129.7	129.5	130.1	129.6	129.9	129.6
*c* ()	44.8	44.9	44.3	44.3	44.8	44.4	44.7
()	111.1	111.4	111.0	110.7	111.2	110.8	111.0
Resolution ()	201.29 (1.331.29)	502.11 (2.192.11)	501.09 (1.121.09)	501.33 (1.361.33)	501.11 (1.131.11)	501.35 (1.401.35)	501.35 (1.401.35)
*R*_merge_ (%)	5.2 (54.7)	3.2 (6.7)	9.4 (49.2)	9.1 (44.0)	10.2 (43.4)	9.1 (33.7)	5.9 (25.5)
*I*/(*I*)	20.7 (1.85)	46.4 (25.7)	13.05 (2.7)	19.0 (4.1)	13.7 (4.0)	23.9 (9.3)	31.1 (9.1)
Completeness (%)	89.2 (90.6)	95.8 (90.5)	97.7 (96.2)	99.5 (100.0)	96.7 (95.4)	99.9 (100)	97.0 (95.4)
Multiplicity	3.7 (3.7)	6.8 (6.2)	4.0 (3.7)	5.0 (4.9)	4.8 (4.6)	7.2 (7.1)	7.4 (7.3)
Refinement							
Resolution ()	201.29		201.09	201.33	201.11	201.35	201.35
No. of reflections	124835	29676	179801	108060	186917	99376	96822
*R*_work_/*R*_free_ (%)	12.28/15.79		12.73/15.17	12.18/15.86	13.33/15.73	13.30/16.75	14.12/17.75
No. of atoms							
Protein	4431		4447	4348	4521	4311	4359
Ligand/ion			24	28	28		2
Water	903		937	914	887	1138	1055
*B* factors (^2^)							
Protein	17.6		12.1	13.6	12.7	12.2	14.1
Ligand/ion			10.4	19.2	13.7		
Water	36.7		31.7	32.1	28.6	40.4	37.7
R.m.s. deviations							
Bond lengths ()	0.006		0.005	0.005	0.005	0.006	0.006
Bond angles ()	1.064		1.049	1.078	1.090	1.035	1.046

**(a):** Substrate specificity of AidH. The activity of AidH towards 3-oxo-C8-HSL was defined as 100%. The structures of the substrates are shown in Fig. 2 ▶.

Substrate	Structure	Activity (%)
C6-HSL	Fig. 2 ▶(*a*)	99.6
3-Oxo-C6-HSL	Fig. 2 ▶(*b*)	97.2
C8-HSL	Fig. 2 ▶(*c*)	98.9
3-Oxo-C8-HSL	Fig. 2 ▶(*d*)	100
C10-HSL	Fig. 2 ▶(*e*)	95.6
3-Oxo-C10-HSL	Fig. 2 ▶(*f*)	97.3
C12-HSL	Fig. 2 ▶(*g*)	89.7
3-Oxo-C12-HSL	Fig. 2 ▶(*h*)	83.3
3-OH-C12-HSL	Fig. 2 ▶(*i*)	91.9
3-Oxo-C14-HSL	Fig. 2 ▶(*j*)	95.4

**(b):** Mutagenic analysis of the functional residues in the active site. 3-Oxo-C8-HSL and C12-HSL were used as substrates. The activity of wild-type AidH was defined as 100%.

	Activity (%)	
Enzyme	3-Oxo-C8-HSL	C12-HSL
WT	100	100
N33G	96.5	92.1
M77G	92.2	80.4
S102G	0	0
L103G	77.2	78.5
F138G	97.3	100
M144G	98.7	82.2
Y160G	0	10.3
M188G	99.2	68.2
F189G	88.8	86.0
F192G	77.8	71.7
E219G	9.1	25.7
F221G	72.7	77.8
H248G	0	0

## Footnotes

1 Supplementary material has been deposited in the IUCr electronic archive (Reference: DW5029). Services for accessing this material are described at the back of the journal.
