# Supplementary material for: High-resolution structures of AidH complexes provide insights into a novel catalytic mechanism for N-acyl homoserine lactonase
Source: Acta Crystallogr D Biol Crystallogr. 2012 Dec 20;69(Pt 1):82–91. doi: 10.1107/S0907444912042369 (PMC3532132; doi:10.1107/S0907444912042369)
Supplement: Supplementary file 1 [file d-69-00082-sup1.pdf]

Supplementary material

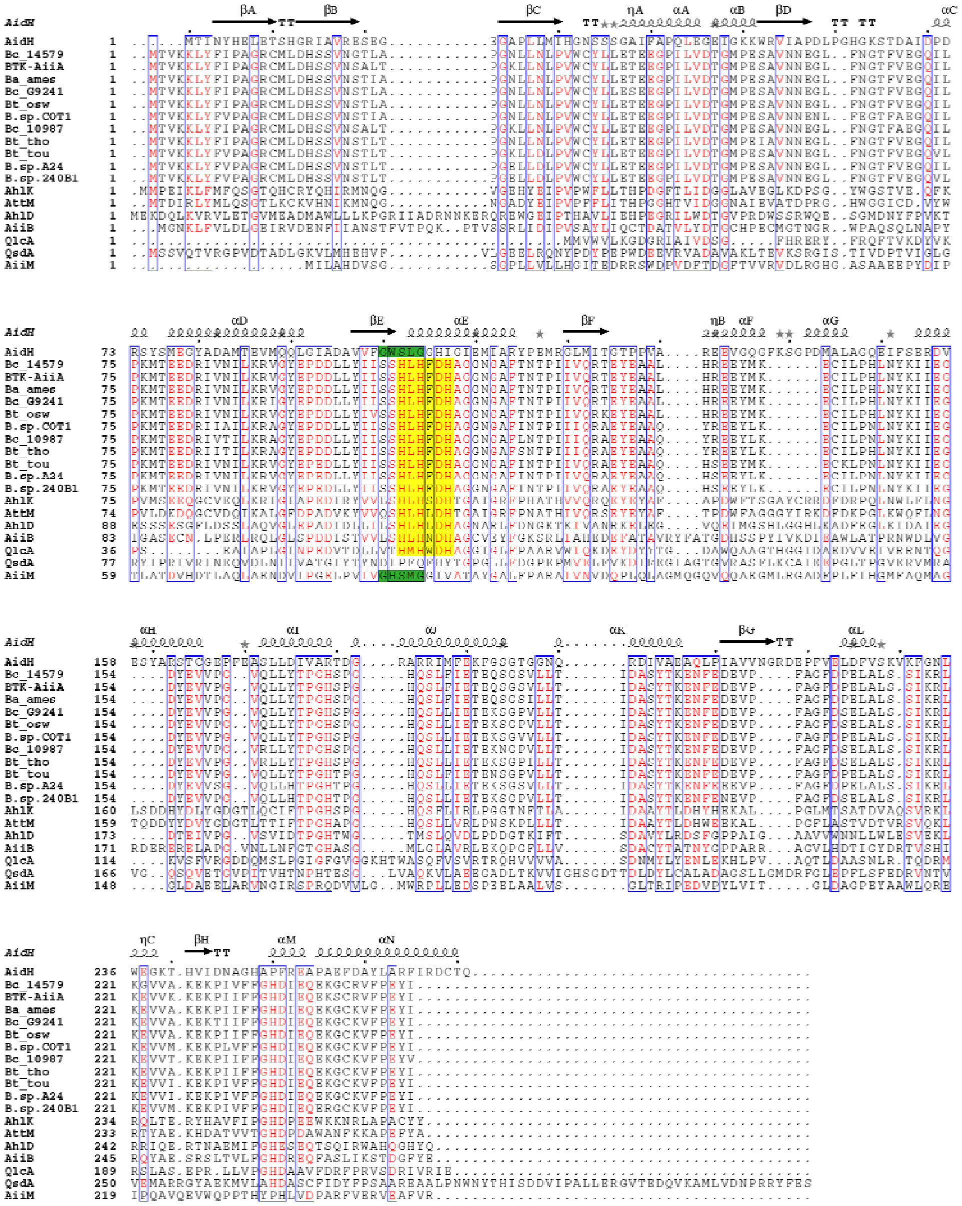

Fig. S1. Amino acid sequence alignment of AidH with other reported AHL-lactonases. The alignment was conducted using ClustalW program. The conserved residues are indicated in red. Areas filled in yellow and green represent motifs 'HXHXDH' and 'GXS/D/CXG', respectively.The lactonases (from top to bottom) are from the following: *Ochrobactrum* sp. strain T63 (AidH, gi:270313530), *Bacillus cereus* 14579 (Bc 14579, gi30021556), *B. thuringiensis serovar kurstaki* (BTK-AiiA, gi22095303), *B. anthracis* Ames strain (Ba ames, gi30263417), *B. cereus* G9241 (Bc G9241, gi47564581), *B. thuringiensis* serovar oswaldocruzi (Bt osw, gi28413776), *Bacillus* sp. COT1 (B. sp. COT1, gi19773593), *B. cereus* ATCC10987 (Bc 10987, gi42738443), *B. thuringiensis* serovar thompsoni (Bt tho, gi22095299), *B. thuringiensis* serovar toumanoffi (Bt tou, gi22095301), *Bacillus* sp. A24 (B. sp. A24, gi21541343), *Bacillus* sp. 240B1 (B. sp. 240B1, gi7416989), *Klebsiella pneumoniae* (AhlK, gi31540969), *Agrobacterium . tumefaciens* (AttM, gi17223785), *Arthrobacter sp.* IBN110 (AhlD, gi31580543), *A. tumefaciens* (AiiB, gi16119885), uncultured Acidobacteria bacterium cosmid p2H8 (QlcA, gi157644500), *Rhodococcus sp.* MP50 (QsdA,gi146742384), *Microbacterium testaceum* StLB037 (AiiM, gi334302761).

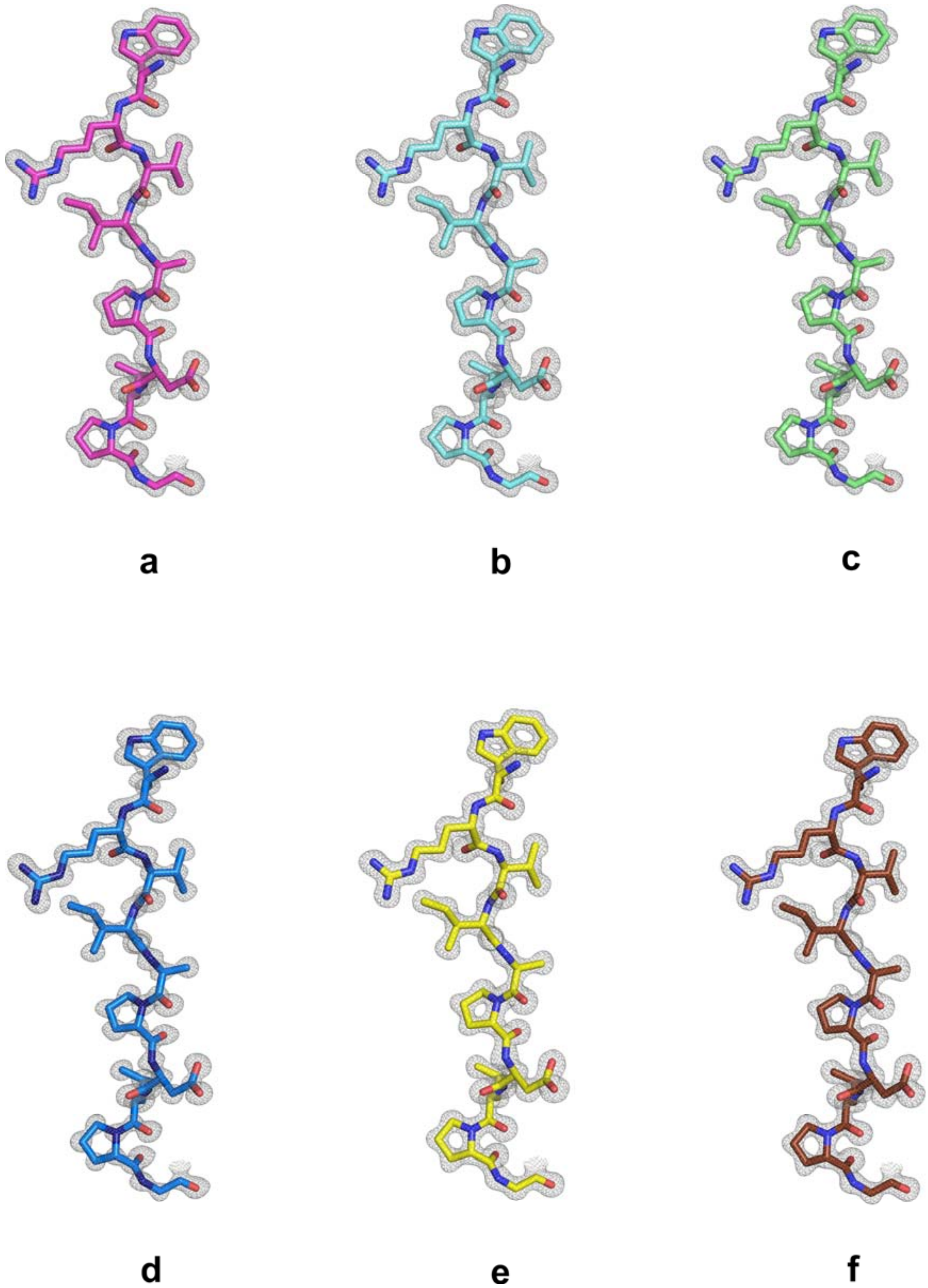

Fig. S2. Representation of electron densities in real space. All the structures referred in this paper have high precision. The 2Fo-Fc electron density for residues (52 to 61) contoured at the 1.5 σ level are shown: (a) wild-type AidH, (b) $AidH_{S102G}$ – C6-HSL complex, (c) AidH – C4-HS complex, (d) $AidH_{E219G}$ – C6-HS complex, (e) $AidH_{S102G}$, and (f) $AidH_{E219G}$.

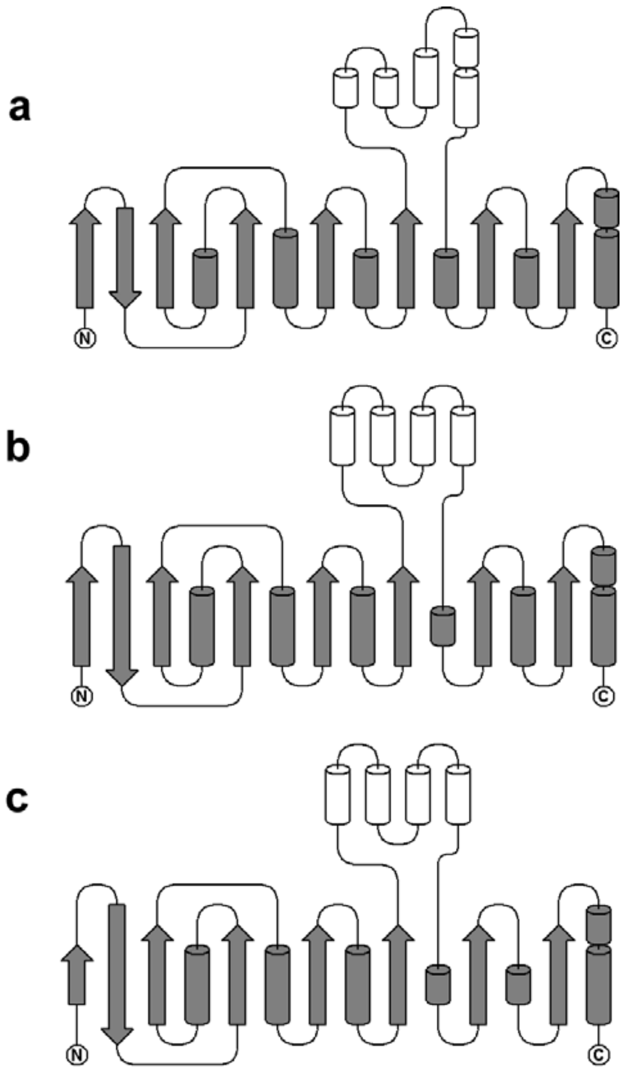

Fig. S3. The topological structures of AidH, 3FOB and 1ZOI. (a) AidH. (b) 3FOB. (c) 2ZOI. The core domain and lid domain are present with gray and white rectangles, respectively.

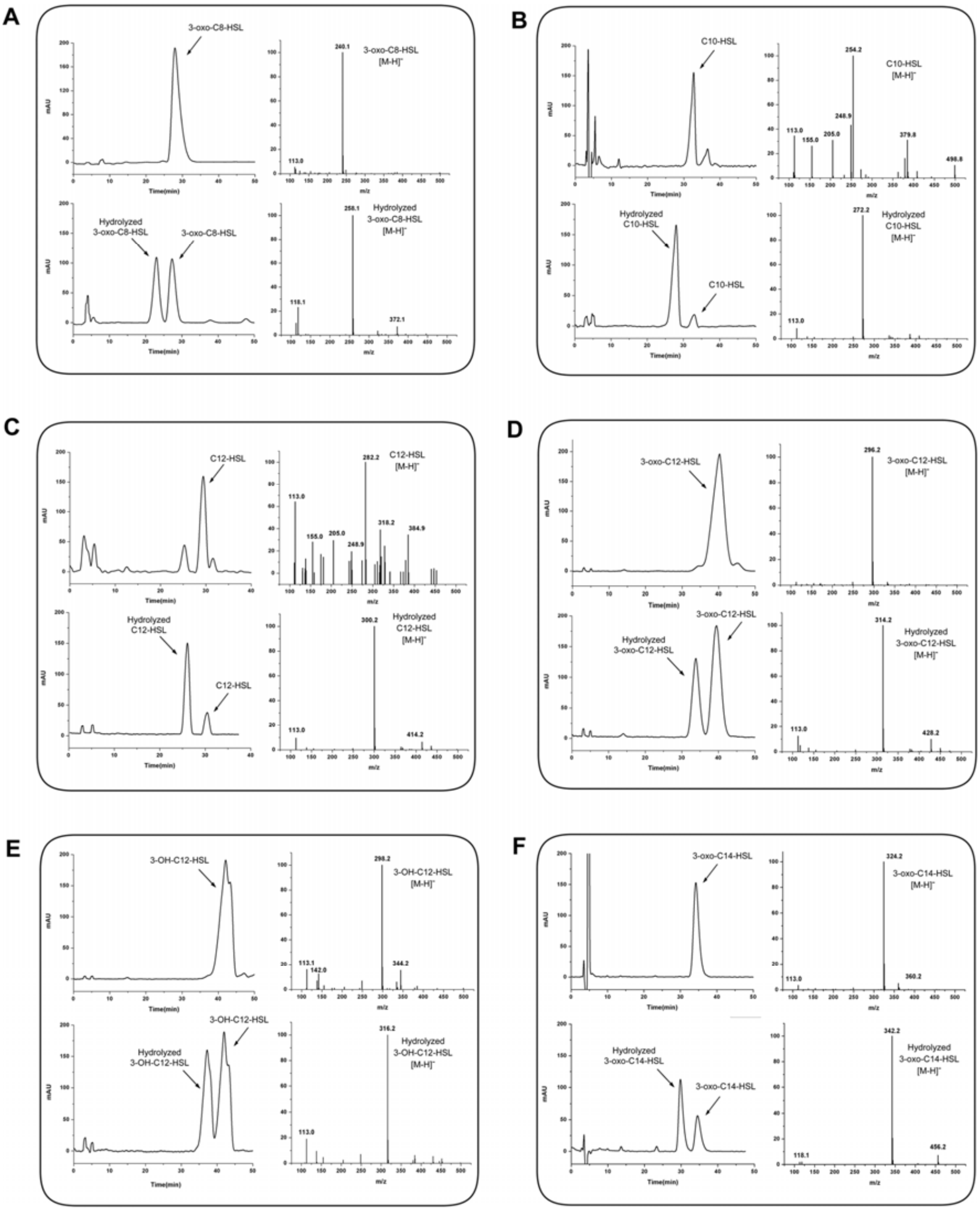

Fig. S4. HPLC and ESI-MS analyses of the AidH-digested products of AHLs with differences in acyl-chain length and substituent at the C3 position. The substrates used for the analysis are 3-oxo-C8-HSL (a), C10-HSL (b), C12-HSL (c), 3-oxo-C12-HSL (d), 3-OH-C12-HSL (e), and 3-oxo-C14-HSL (f). HPLC profiles of the substrate alone and substrate treated with AidH are shown in the left-top and left-bottom, respectively. The peaks corresponding to the substrate and product are indicated by arrows. Electrospray ionization spectral profiles of the substrate and product are shown in the right-top and left-bottom, respectively. We labeled the comparable peaks with their respective m/z.

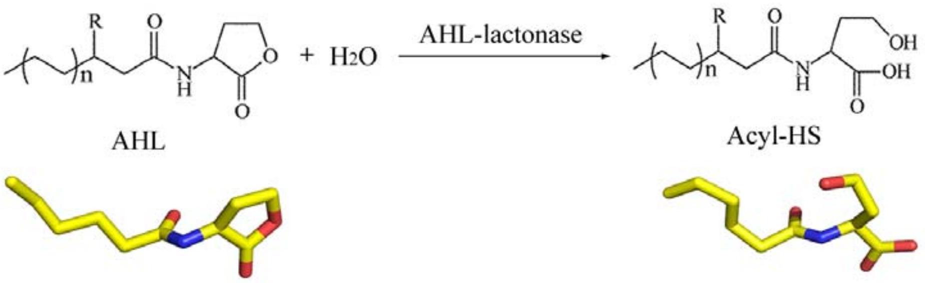

Fig. S5. Scheme of the reaction catalyzed by AHL-lactonase. AHL-lactonase catalyzes the conversion of AHL (*N*-acyl homoserine lactone) to AHS (*N*-acyl homoserine).

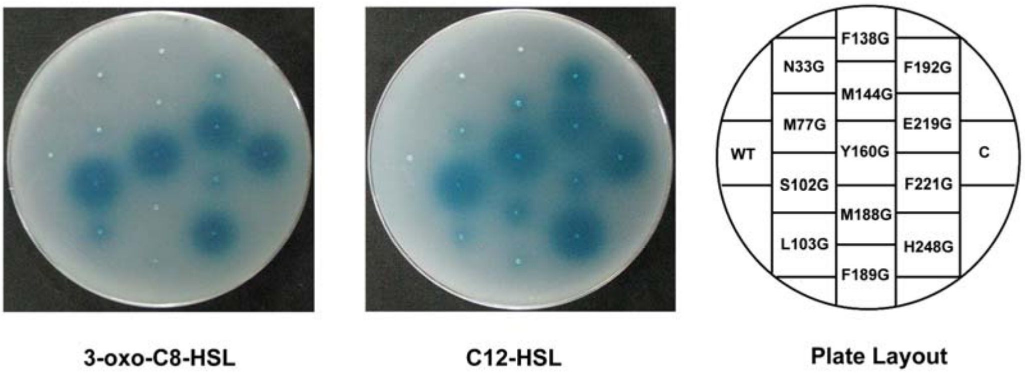

Fig. S6. Enzymatic activity assay. The activity of wild-type AidH and different mutants against 3-oxo-C8-HSL (left) and C12-HSL (middle) is indicated by the size of the blue-circle in the NTL4 plate. Note that the mutants S102G, Y160G, E219G, and H248G are almost inhibited from degrading AHLs. The activity of mutants M188G, F189G, F192G, and F221G decreased to a certain degree. WT indicates the wild-type AidH, whereas C indicates the control (sample without treatment by enzyme).

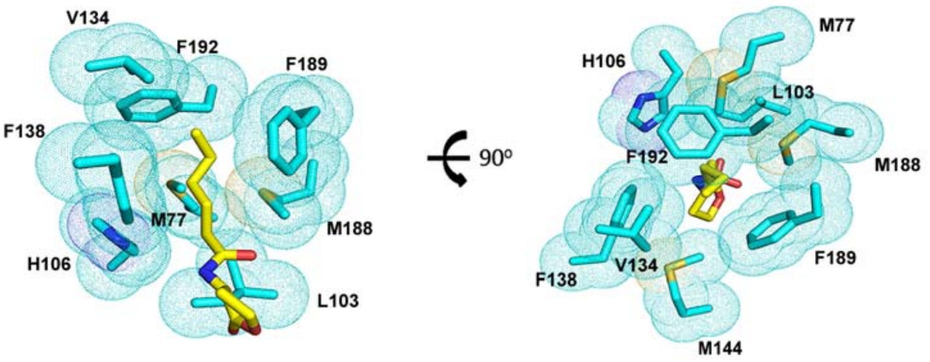

Fig. S7. Structural details of C6-HSL binding in the substrate-binding tunnel. The hydrophobic residues of the tunnel are highlighted in the “dotted” van der Waals radius representation.

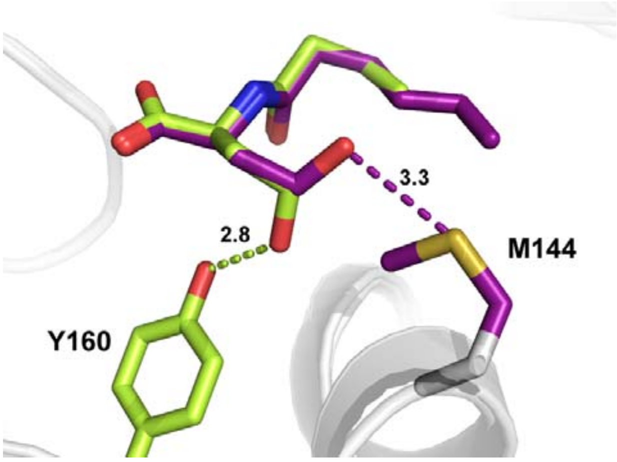

Fig. S8. Two possible conformations of the hydroxyl group of the product. In the AidH–C4-HS complex (lemon), the hydroxyl group of C4-HS forms a hydrogen bond with Tyr160. In the $\mathrm{AidH}_{\mathrm{E219G}}$–C6-HS complex (purple), the hydroxyl group flips to the opposite side and forms a hydrogen bond with Met144. Hydrogen bonds are depicted as short-dashed lines

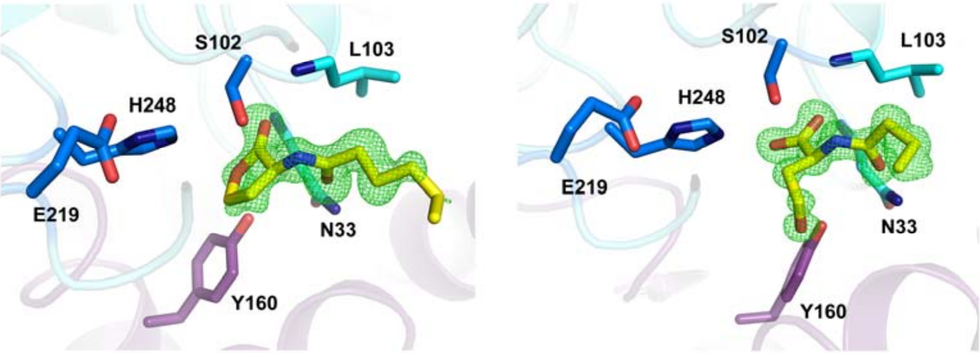

a b

Fig. S9. Substrate and product binding with AidH. (a) Active site of the $\mathrm{AidH}_{\mathrm{S102G}}$–C6-HSL (enzyme–substrate) complex. (b) Active site of the wild-type AidH–C4-HS (enzyme–product) complex. The OMIT Fo-Fc electron density of C6-HSL and C4-HS contoured at 4.5 σ level is shown in green mesh.

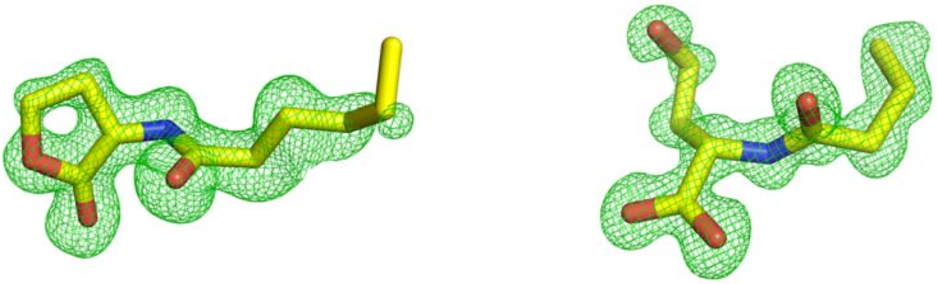

a b

Fig. S10. OMIT map of C6-HSL (a) and C4-HS (b). The OMIT Fo-Fc electron density of C6-HSL and C4-HS contoured at the 4.5 σ level is shown in the green mesh.
